# Editorial: Erythropoietin and Its Analogues as Therapeutics for Neurological Diseases

**DOI:** 10.3389/fphar.2022.841538

**Published:** 2022-02-14

**Authors:** Michael Brines, Stephana Carelli, Michele Samaja, Edith M. Schneider Gasser

**Affiliations:** ^1^ Feinstein Institutes for Medical Research, Manhasset, NY, United States; ^2^ Pediatric Research Center “Romeo ed Enrica Invernizzi” Foundation, Milano, Italy; ^3^ University of Milan, Italy, and MAGI Group, San Felice Del Benaco, Brescia, Italy; ^4^ University of Zurich, Zürich, Switzerland

**Keywords:** EPO receptor, metabolism, iron, hypoxia, inflammation, hypercapnia

## Introduction

### Background

Erythropoietin (EPO), a glycoprotein produced and released by kidney interstitial fibroblasts, is well known since 1905 to stimulate erythropoiesis in the bone marrow (Carnot and Deflandre, 1906). EPO was purified in 1977 (Miyake et al., 1977) and recombinant human EPO (rhEPO) was successfully used to correct anemia in 1987 (Eschbach et al., 1987). The EPO synthesis rate depends on hypoxia, and the discovery of the mechanisms whereby oxygen regulates EPO production worth the 2019 Nobel Prize in Medicine to Gregg L. Semenza, Peter J. Ratcliffe and William Kaelin Jr. EPO was recently recognized to play an additional crucial role in the central nervous system. In response to brain damage and inflammation, astrocytes, microglia, neurons, pericytes, and endothelial cells synthesize and release EPO which binds to EPO receptors (EPOr) expressed by multiple tissues/cells, but especially neurons. Signaling through EPOr activates JAK-2, which turns on the STAT, the PI3K/Akt and the Erk pathways to provide protection via attenuation of inflammation and neuronal apoptotic and death processes, while also triggering tissue repair mechanisms. In the nervous system EPO release can be modulated by stimuli such as hypoxia, inflammation, and iron handling alterations. A key step is the qualitative and quantitative distribution of the EPOr isoforms in the target cells at specific developmental stages, which enabled the development of non-erythropoietic analogues of EPO that are neuroprotective but do not stimulate erythropoiesis, thus avoiding undesired outcomes such as increased blood viscosity or risk of thrombosis. Some of these EPO analogues are currently being evaluated for clinical use.

## Aims of the Special Issue

**FIGURE 1:**
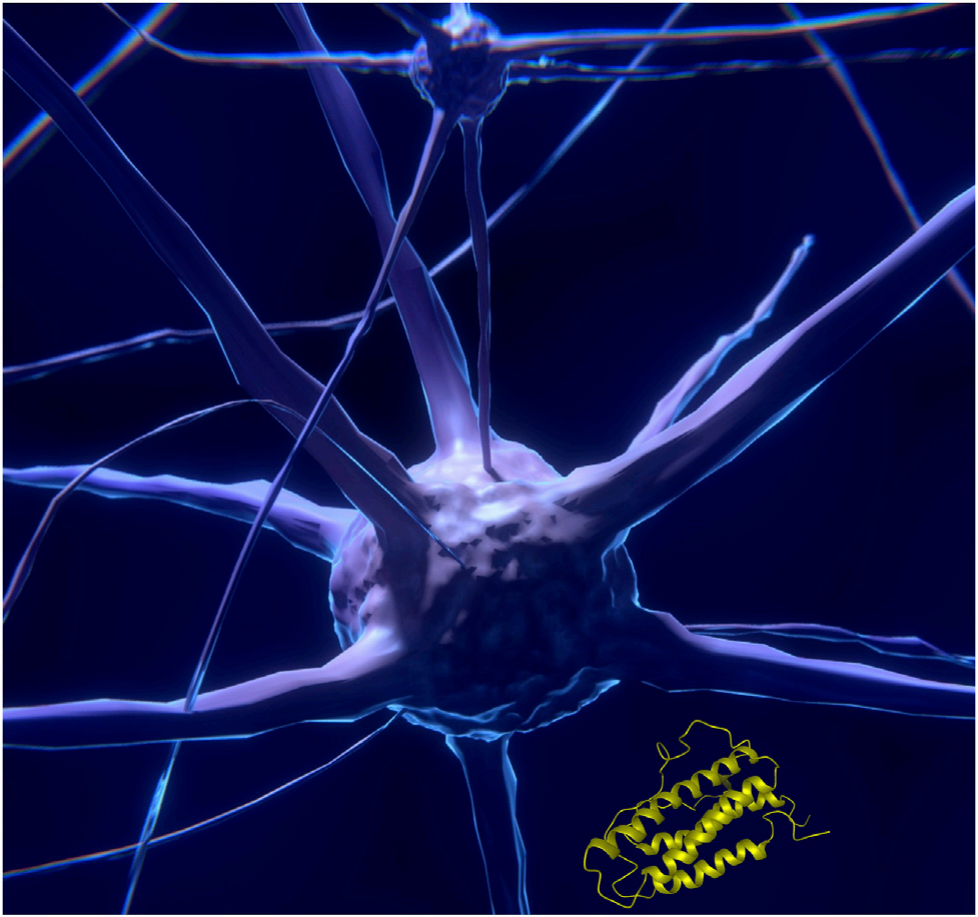
The neuron network and logo of the present special issue. Kind concession of Pixabay, Author Colin Behrens.

The aim of this research topic was to foster an interdisciplinary exchange of the advances in knowledge concerning EPO, its analogues and derivatives, receptor isoforms and target cells in the nervous system (Figure 1). We were confident that investigating the mechanisms by which EPO and its analogues affect the nervous system, we would be able to further refine therapeutic strategies aimed at treating neurological diseases. Indeed, continued multidisciplinary research in this area at several levels, from chemistry to neuropathology, has led to a more comprehensive understanding of neurobiology in health, as well as of the nervous system responses to injury and neuropathological processes.

## How the Contributions met Our Expectations

Dey et al. review the effects of EPO on non-hematopoietic tissues and the mechanisms underlying metabolic regulation. Although they mainly focused upon the adipose tissue response to obesity-inducing diets, numerous issues raised concern the role of brain in this regulation. In fact, while EPO treatment may reduce fat mass and body weight by improving glucose utilization, within the brain the hypothalamus, a master regulator of appetite and energy expenditure, plays a key role. The Authors discuss a critical role for leptin, a Type 1 cytokine that belongs to the same superfamily of EPO. Interestingly, both EPOr and leptin receptors are localized in the same proopiomelanocortin producing neurons, which could suggest considerable functional overlap of the two cytokines. The Authors further discuss the sexual dimorphism of the regulation of metabolism by brain EPO and support the view that EPO/EPOr may have substantial benefits on human health by finely regulating the metabolism.

Ottolenghi et al. review the potential use of EPO to reduce hypoxemic neurological damage in neonates with congenital heart defects. By downregulating the oxygen delivery to neurons, this devastating pathology challenges brain development though disruption of the equilibrium between redox imbalance and antioxidant defense, thereby causing irreversible brain damage and impairing brain development. Although representing a clinical therapy to counteract fetal hypoxia, maternal hyperoxygenation is not without risks. Thus, studies are under way to evaluate the potential role of rhEPO as neuroprotective factor based not only on its antioxidant properties, but also on the promotion of oligodendrocytes differentiation/maturation and stimulation of myelin production. Concerns remain as to route of EPO administration because the poor permeability across the blood-brain barrier requires high doses that may lead to excessive erythropoiesis.

The critical issue of the EPO doses to be used clinically without causing adverse erythropoietic effects is the main objective of the original study reported by Arias-Reyes et al.. The Authors examine in rodents the dose-dependent impact of EPO in the carotid body (CB) response to hypoxia or hypercapnia, based on a controversial data previously obtained, which showed that high EPO doses failed to stimulate central nervous system discharges as compared to the opposite by lower doses. The Authors found that EPO produces a dual effect on CB chemoreception: at low doses (<0.5 IU/ml) EPO stimulates the hypoxic response to hypoxia, while at higher doses (>1 IU/ml) EPO inhibits central nervous system activity via increased nitric oxide (NO) production by type I CB cells. Besides representing an important caution when treating hypoxemic patients, these findings are particularly relevant because they suggest a critical interaction between EPO and the activity of NO synthases, especially the endothelial one, which may be particularly relevant in populations residing at high altitude and therefore under continuous erythropoietic stimulation.

Finally, Newton et al. critically review the role of EPO and EPO derivatives in cognition. Besides providing beneficial effects in several psychiatric disorders (schizophrenia, bipolar disorders, and major depression), EPO appears as the only drug capable of delaying cortex thinning in schizophrenia. The mechanisms underlying these benefits encompass changes in gene expression, improvement of neurogenesis, synaptic plasticity, oligodendrogenesis, and anti-inflammatory activity. Remarkably, an EPO non-erythropoietic analogue, carbamoylated EPO, apparently elicits the same favourable effects elicited by EPO, but without increasing erythropoiesis. The Authors describe a stereochemical model that compares the erythropoietic vs. non-erythropoietic receptor signalling whereby the JAK2/STAT signalling is engaged by EPO only, but the PI3K-Akt and ERK signalling are engaged by both.

## Conclusion

The articles published in this Special Issue aimed at assessing the potential role of EPO and its analogues in driving neuroprotection in acute injury and neurodegenerative diseases. Remarkably, these contributions are based on basic or clinical data in the perspective of multidisciplinary efforts “from bench to bed—and return”. Clearly, to get a full and exhaustive picture of the role of EPO in neurological disorders, much work is still required to address the following topics:

• Further dissection of the roles of EPO in normal and pathologic neurobiology and neurodevelopment.
• Dissecting the biological activities and molecular mechanisms underlying the EPO neuroprotective effects.
• Dissecting EPO’s effect on cell metabolism as therapeutic target.
• Identifying EPO analogues that act as potential therapeutic treatments in CNS.
• Assigning roles for specific cell-type EPO receptor isoforms.
• Elucidating which acute pathologies (e.g., spinal cord injury, perinatal brain injury) or chronic neurodegenerative diseases (e.g., Parkinson disease, amyotrophic lateral sclerosis, Huntington disease, and psychiatric conditions) may be targetable for rhEPO (or EPO analogues)-based therapies.
• Determining effects of EPO/analogues on cognitive function/depression/schizophrenia.
• Identifying specific stimuli, e.g., hypoxia, hyperoxia, brain hypoxia and ischemia, inflammation, iron dysfunction, oxidative/metabolic stress, which may have potential positive or negative interference with EPO-based mechanisms.
• Introducing novel multidisciplinary experimental models aimed at covering the gap between basic mechanisms and clinical outcomes.
• Assessing potential clinical advances (e.g., trials in peripheral neuropathy).
